# Metabolic Brain Network Analysis of FDG-PET in Alzheimer’s Disease Using Kernel-Based Persistent Features

**DOI:** 10.3390/molecules24122301

**Published:** 2019-06-21

**Authors:** Liqun Kuang, Deyu Zhao, Jiacheng Xing, Zhongyu Chen, Fengguang Xiong, Xie Han

**Affiliations:** 1 School of Data Science and Technology, North University of China, Taiyuan 030051, China; 1307084309@st.nuc.edu.cn (D.Z.); hopenxfg@nuc.edu.cn (F.X.); 2 School of Software, Nanchang University, Nanchang 330047, China; xingjiacheng628@163.com; 3 School of Software, East China Jiaotong University, Nanchang 330013, China; 15079561913@139.com

**Keywords:** Alzheimer’s disease (AD), network measure, graph theory, brain network, positron emission tomography (PET), persistent homology

## Abstract

Recent research of persistent homology in algebraic topology has shown that the altered network organization of human brain provides a promising indicator of many neuropsychiatric disorders and neurodegenerative diseases. However, the current slope-based approach may not accurately characterize changes of persistent features over graph filtration because such curves are not strictly linear. Moreover, our previous integrated persistent feature (IPF) works well on an rs-fMRI cohort while it has not yet been studied on metabolic brain networks. To address these issues, we propose a novel univariate network measurement, kernel-based IPF (KBI), based on the prior IPF, to quantify the difference between IPF curves. In our experiments, we apply the KBI index to study fluorodeoxyglucose positron emission tomography (FDG-PET) imaging data from 140 subjects with Alzheimer’s disease (AD), 280 subjects with mild cognitive impairment (MCI), and 280 healthy normal controls (NC). The results show the disruption of network integration in the progress of AD. Compared to previous persistent homology-based measures, as well as other standard graph-based measures that characterize small-world organization and modular structure, our proposed network index KBI possesses more significant group difference and better classification performance, suggesting that it may be used as an effective preclinical AD imaging biomarker.

## 1. Introduction

Alzheimer’s disease (AD) is one of the most common neurodegenerative neurological diseases and is the most common form of dementia in the elderly [1]. Its clinical manifestations include long-term memory loss, cognitive decline, language disorders, and other symptoms. AD seriously affects the normal life of the elderly. However, the pathology of AD is not yet clear [1]. Some existing imaging technologies are used to explore the mechanisms of human brain function. Compared to magnetic resonance imaging (MRI), fluorodeoxyglucose positron emission tomography (FDG-PET) has been demonstrated to be a more precise predictor of both AD and mild cognitive impairment (MCI), and is more suitable for monitoring disease progression [2]. It collects and measures changes in glucose metabolism values in brain regions or local brain cells. The signals are then converted into effective three-dimensional images and the connectivity between brain regions are analyzed.

The topological organization of metabolic brain networks have been successfully characterized in many cases using various measures based on graph theory [3,4,5], such as characteristic path length (CPL) [6], global efficiency [7], modularity (Mod) [7,8], and network diameter (ND) [9], to name but a few. Specifically, in patients with AD and MCI, several research groups have reported topological alterations in the whole-brain connectome, including a loss of small-worldness [10], a redistribution of hubs [9,11], and a disrupted modular organization [12]. Traditionally, weighted networks usually require defining a set of thresholding values before quantifying network topology [13,14,15], which may result in inconsistent network features when the thresholding values vary. Generally, the choice of threshold is rather arbitrary and there are no widely accepted criteria [16,17].

Recently, persistent homology [18] in algebraic topology has been studied to detect persistent structures generated over all possible thresholds [19,20,21,22,23] in brain network analysis. There have been significant efforts to model evolution of brain networks and to link network topology to network dynamics. This method constructs multiscale network for all possible thresholds wherever the persistent topological features over the evolution of the network changes are identified. Its ability to handle noisy data and provide homological information has turned it into a successful tool for the analysis of brain network structures [24]. One typical application of persistent homology is in a Betti number plot (BNP) [18], which has been successfully applied to the brain network research on epilepsy [20], autism spectrum disorder, and attention-deficit hyperactivity disorder [19,23]. As BNP ignores the association between persistent features and forthcoming thresholding value changes, we proposed an integrated persistent feature (IPF) by integrating an additional feature of connected component aggregation cost with BNP, and applied it to measure an AD network using resting state functional MRI (rs-fMRI) in our prior study [25]. However, both BNP and IPF applied linear regression analysis for computing the slope of the plot over all thresholds as a univariate network index. Such a slope-based approach may not accurately characterize the changes of persistent features over graph filtration because the curves are not strictly linear. Moreover, our previous IPF works well on an rs-fMRI cohort though it has not been used to study metabolic brain networks yet.

In this paper, we borrow the idea of kernel methods [26,27] on persistent homology and propose a kernel-based IPF (KBI) index based on our prior work on IPF. We hypothesized that our KBI index may help to better reveal the difference between brain networks. With the cross-sectional FDG-PET imaging data of 140 AD, 280 MCI, and 280 normal control (NC) individuals, we set out to test this hypothesis by computing the KBI indices that measure the differences between AD, MCI, and NC groups. We further perform statistical inference and classification to validate the power of KBI.

## 2. Results

In this section, we use FDG-PET data to evaluate statistical power and classification performance of our proposed KBI index for the analysis of brain metabolic networks related to AD. We further compared it with prior persistent features, BNP [19,21,23] and SIP [25], as well as some other standard graph-based indices.

### 2.1. Metabolic Brain Networks

**Figure 1:**
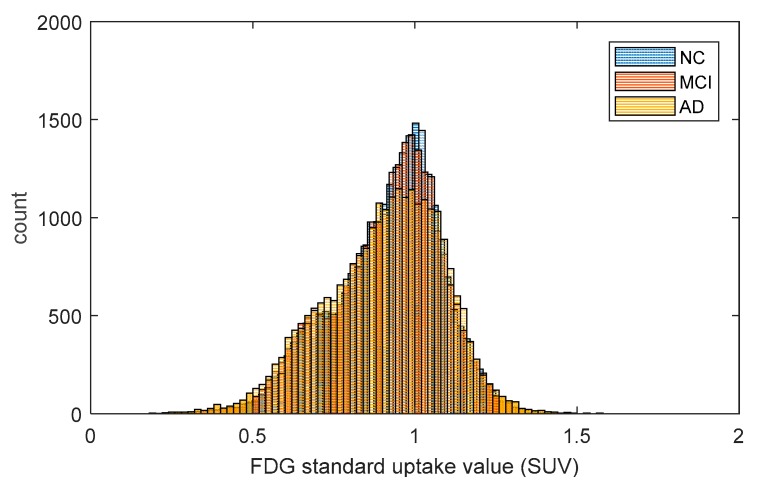
The fluorodeoxyglucose (FDG) uptake distribution of AD, MCI, and NC groups.

**Figure 2:**
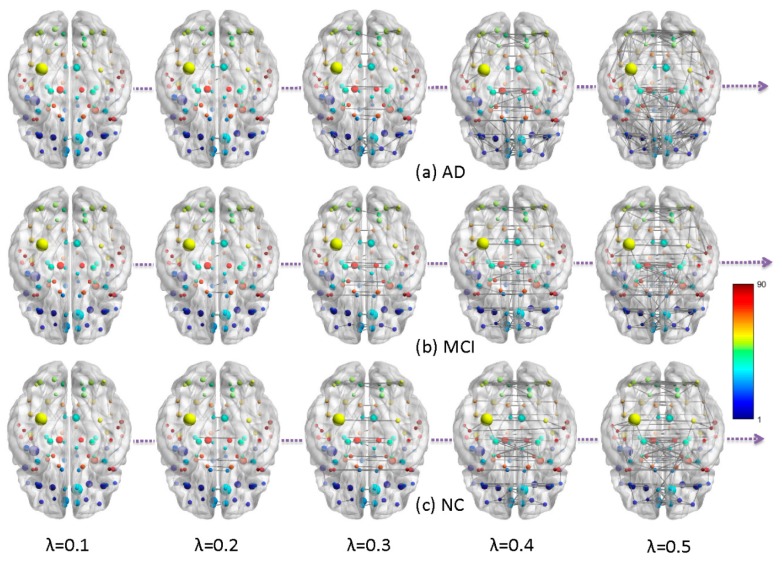
The constructed multiscale networks of AD, MCI, and NC by graph filtration λ, and the node color represents the ROI index predefined in AAL-90 atlas.

After data preprocessing, the summarized point cloud were extracted from PET 3D imaging using predefined automated anatomical labeling atlas with 90 (AAL-90) regions of interests (ROI) [28]. We obtained the SUV matrix for all 700 subjects in all 90 ROIs and plot three histograms, in Figure 1, to show the global distributions of FDG uptake in AD, MCI, and NC. As the number of AD is half of MCI and NC, we have normalized the SUV distribution of AD by doubling its statistics. We observed that the AD cohort has lower glucose metabolism than MCI and NC, but no significant differences were detected in the statistical inference of permutation test. We calculated the Pearson-based correlation distance of FDG uptake between each pair of brain regions using Equation (1) and constructed group-wise brain metabolic networks. The three multiscale networks of AD, MCI, and NC groups are shown in Figure 2, which visualizes the evolution of brain networks over different thresholds.

### 2.2. Brain Network Features

**Figure 3:**
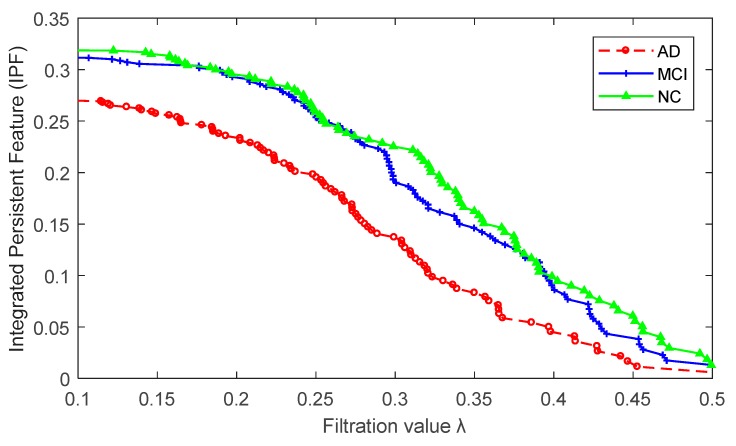
The proposed integrated persistent feature (IPF) plot for three group-wise networks of AD, MCI and NC, respectively.

We computed the values of graph-based network indices (CPL, ND, and Mod) in three groups based on their weighted networks after filtering their edges, whose corresponding *p*-values passed a statistical threshold (Bonferroni corrected *p* < 0.05). We then obtained the multiscale network according to graph filtration and computed the BNP and IPF index (i.e., SIP), as well as KBI index. Figure 3 shows three separate IPF plots of AD, MCI, and NC. All brain network index values are shown in Table 1. The differences between groups need to be further verified by statistical inference and classification.

### 2.3. Statistical Group Difference Performance

In this study, we use the permutation test for 10,000 permutations between any two groups, and show the resulting *p*-value in Table 2. Only the proposed KBI index obtained a significant difference in any between-group at the significance level of 0.05.

### 2.4. Classification Performance

**Figure 4:**
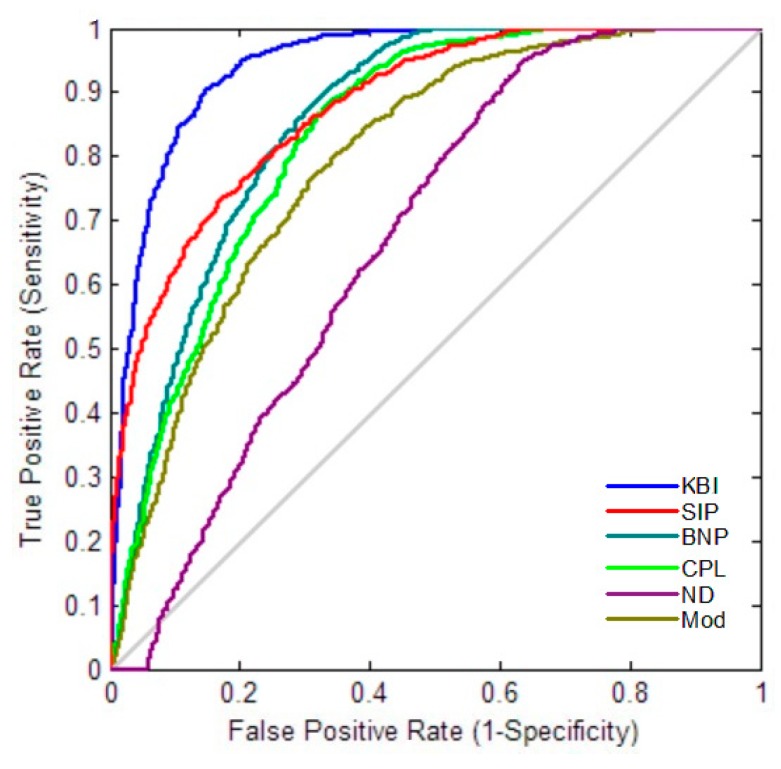
Comparison of ROC curves of different network indices for MCI vs. NC.

**Figure 5:**
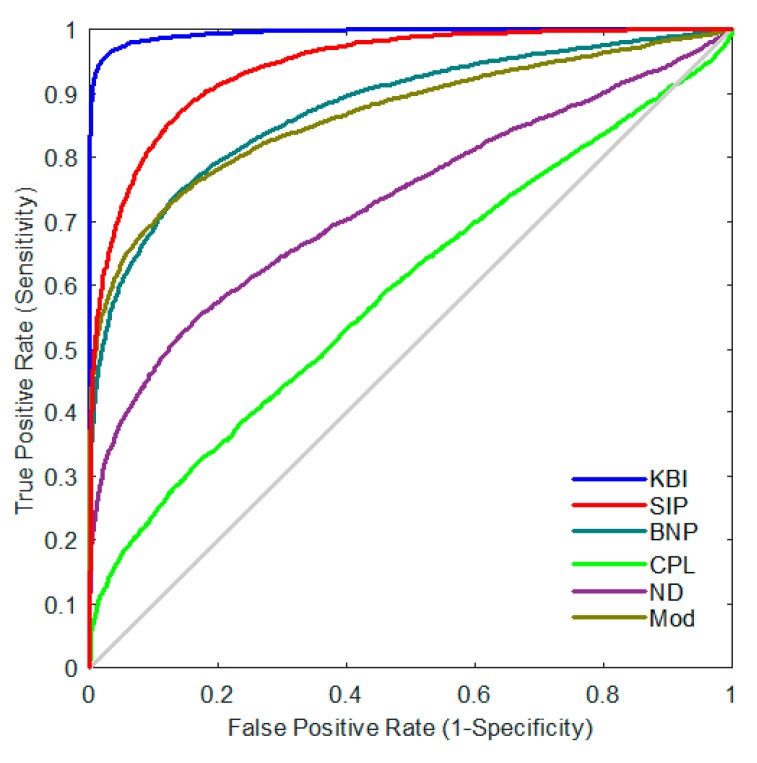
Comparisons of ROC curves of different network indices for AD vs. NC.

**Figure 6:**
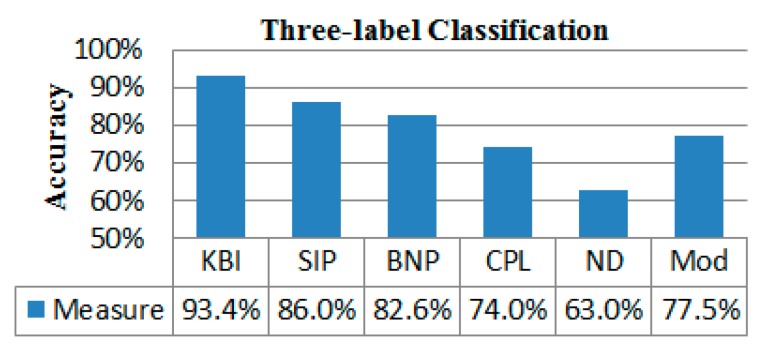
Three-label classification.

Furthermore, we resampled the networks 5000 times for each group with the resampling rate of 0.5, and obtained 5000 values of each network index for each group. We then performed leave-one-out crossvalidation to evaluate the classification powers of two-label (Figure 4 and Figure 5) and three-label (Figure 6) by SVM. Our KBI shows better classification performance than prior persistent features, SIP and BNP, as well as other standard graph-based features, including CPL, ND, and Mod.

## 3. Discussion

### 3.1. Present Findings

This study has three main findings.

First, from Figure 1, we found that the AD cohort has lower glucose metabolism than MCI and NC. This may imply cognitive impairment in AD and MCI. Such an inference is further partly confirmed by graph theory analysis because a larger CPL is present in AD and MCI, while the network with smaller CPL is considered to be efficient.

Second, in our previous study [25], we had developed a univariate network index, SIP, based on homology to model graph dynamics over all possible scales and applied it to study the rs-fMRI data of AD. We found the SIP values of AD were lower than MCI and much lower than NC. In the current PET data, we still find the SIP values show the same pattern AD < MCI < NC, suggesting a slower network integration rate in AD and MCI groups. Thus, the results from both independent cohorts provide consistent empirical evidence for decreased functional integration in AD dementia and MCI.

Finally, we propose a novel univariate network index KBI to enhance our previous study based on persistent homology. With our univariate KBI index, the difference of persistent features between cognitive dysfunction and NC brain network can be measured more accurately. Our preliminary experimental results demonstrate that the proposed KBI may greatly boost prior SIP and BNP power in both statistical inference and classification analyses. The KBI also outperforms other standard graph-based methods, such as CPL, ND, and Mod, suggesting that our method may serve as a valuable preclinical AD imaging biomarker.

### 3.2. Exploring Other Connectivity Definitions

There are many types of distance functions to construct weighted networks in brain network analysis [29], such as Pearson correlation, partial correlation, psycho–physiological interactions, ReHo, partial least squares, wavelet-based correlation, mutual information, synchronization likelihood, principal component analysis, independent component analysis, cluster analysis, dynamic causal modeling, Granger causality modeling, structural equation modeling, and multivariate autoregressive modeling, among others. At present, it is difficult to put forward the evaluation criteria of these methods, and few studies have compared them comprehensively. Although the Pearson correlation that we used in this study may be the most practical scheme to define the connectome in AD studies, there is still debate about the choice of connectivity definition [29,30]. Therefore, we performed four other connectivity definitions to explore more potentials in defining connectivity network. They were Kendall correlation [31], Spearman correlation [32], partial least squares [33], and Granger causality modeling [34]. The obtained *p*-values of our proposed KBI with these distance functions are shown in Table 3. There was no significant difference if Granger causality modeling was used, while the other three methods detected at least one significant difference. It should be noted that none of these methods performed significantly better than Pearson correlation (Table 1) in our current dataset. Moreover, when we checked all measures to discriminate AD, MCI, and NC by these methods, we found that the three-label classification accuracy of BNP (88.3%) was improved greatly if the partial least squares method was applied, while the performances in other cases have not been improved significantly. Such an empirical study may justify the connectivity definition adopted in our current work.

### 3.3. Ways of Network Construction

Graph-based brain connectome analyses are sensitive to the choice of parcellation schemes. To assess the effects of different parcellation strategies, we carried out the same set of analyses with another commonly employed atlas, the Harvard–Oxford atlas [35,36] with 110 ROIs (HOA-110). The detailed statistical significances of between-group difference on HOA-110 are presented in Table 4. Again, our proposed KBI achieved better statistical power.

In the metabolic network construction, the common practice is building a group-wise brain network for each group as there is only one summarized value (average SUV) in each ROI. However, we notice that some studies [37] constructed subject-wise networks by dividing each ROI of a subject into blocks to obtain the correlation distance between any two ROI. Thus, subject-wise networks were constructed. We did not study this method in as it would require additional discussion that would exceed the scope of this paper.

In addition, we defined the connectivity between two brain regions as 1-Pearson correlation in Equation (1) in our study. Although some existing studies [19,23] also define such kinds of connectivity in analyzing brain network properties, the common practice in brain network analysis based on graph theory is to directly specify the Pearson correlation as the edge weight. To assess the effect of different connectivity definitions on other graph-based measures, we performed statistical inference on the brain networks whose edges were defined as directly based on Pearson correlation, and the statistical *p*-values of graph-based measures are shown in Table 5. We found the results were different from the previous results in Table 2, suggesting that graph-based measures could be affected by way of connectivity definition. We also found that none of the graph-based measures could detect all between-group differences significantly in either connectivity definition.

### 3.4. Limitations and Future Work

Despite the promising results obtained by applying our proposed network index KBI based on persistent homology to PET, there are three important caveats. First, the current study only takes the zeroth persistent homology into account. Higher-order persistent features are also worth studying. In future, we will try to improve the performance of our method by considering higher dimensional persistent homology, such as the first Betti number, which is designed to calculate the number of holes in a graph and may boost the performance, especially in sparse networks that tend to have more holes. Then, although the subject-wise network is more convenient, efficient, and useful than group-wise network for brain network analysis, as we discussed in our prior study [25], we only measured the group-wise metabolic brain network according to regular practice in PET data analysis. In future, we will validate the KBI in a subject-wise metabolic network. Finally, this study was based on cross-sectional PET analysis, and we compared their network indices. With longitudinal PET analysis, we may further study the evolution between longitudinal brain networks by quantifying the difference of their persistent features.

## 4. Materials and Methods

**Figure 7:**
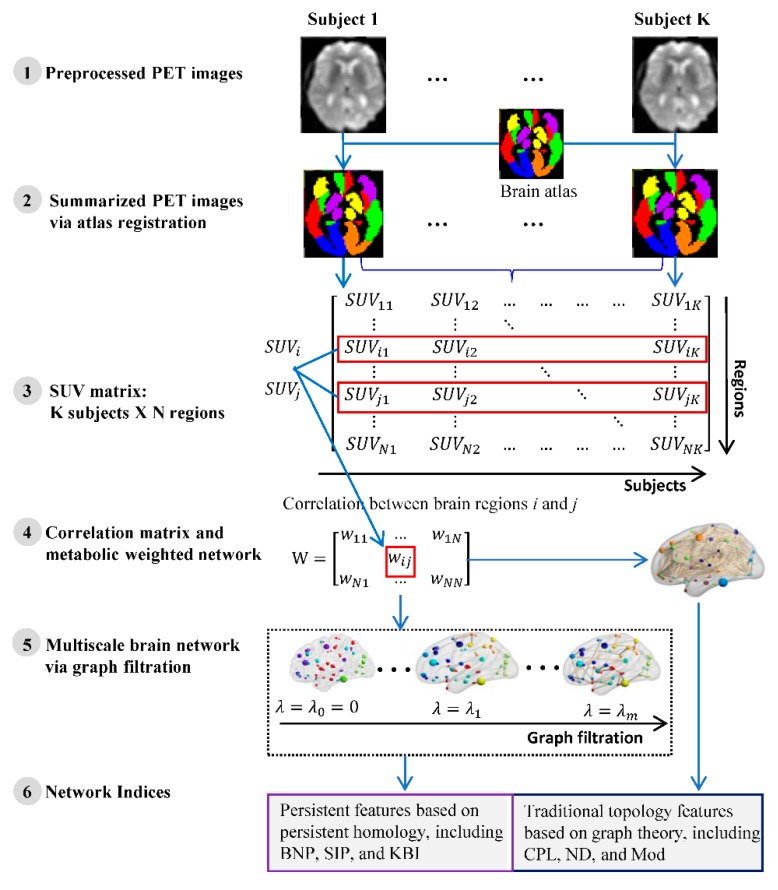
The pipeline of the brain network analysis based on a group of subjects. SUV, support vector machine; BNP, Betti number plot; SIP, slope of IPF plot; KBI, kernel-based IPF; CPL, characteristic path length; ND, network diameter; Mod, modularity.

Figure 7 shows the pipeline of our framework, where the data flow from FDG-PET brain images to some network indices. The details are described in following subsections.

### 4.1. Participants

Data used in the preparation of this article were obtained from the Alzheimer’s Disease Neuroimaging Initiative (ADNI) database (adni.loni.usc.edu) [38,39]. ADNI was launched in 2003 as a public–private partnership led by Principal Investigator Michael W. Weiner, MD. The primary goal of ADNI has been to test whether serial magnetic resonance imaging (MRI), positron emission tomography (PET), other biological markers, and clinical and neuropsychological assessment can be combined to measure the progression of mild cognitive impairment (MCI) and early Alzheimer’s disease (AD).

In this study, we chose 700 subjects with FDG-PET data from ADNI2. To match the three research cohorts of AD, MCI, and NC in gender and age, 140 AD, 280 MCI, and 280 NC subjects from 57 sites across North America were selected. The detailed cohort information is described in Table 6.

### 4.2. FDG-PET Data Acquisition and Preprocessing

All FDG-PET scans were obtained using Siemens, GE, and Philips PET scanners. Details of the PET data acquisition is described at http://adni.loni.usc.edu/methods/pet-analysis/pre-processing/. All FDG-PET scans used in this research are preprocessed (step 1 of Figure 7) as follows [40]. First, in order to eliminate the individual differences in brain morphology between subjects such that they can completely coincide and be subject to effective statistical analysis, we used the software toolkit Statistical Parametric Mapping (SPM8) [41] in MATLAB (Mathworks Inc, Natick, MA, USA) to linearly align the images into the Montreal Neurological Institute (MNI) space using the TPM.nii template file released with SPM. Second, we borrow a brain mask from SPM, exclude the brain stem and only keep the cerebral cortex (because the cerebral cortex is the object of this study), and then segmented all the images with this cerebral mask. Third, we conducted spatial smoothing with a Gaussian kernel of the full width at half maximum (FWHM) equal to (8,8,8) in three directions (x,y,z) to improve signal-to-noise.

### 4.3. Network Construction

A weighted graph is a natural and efficient way to represent metabolic brain network because it represents a discretized version of original PET images. In computer graphics, polygon meshes, as a class of graphs with particular connectivity restrictions, are extensively used to represent the topology of an object [42]; however, the mesh representation may not be the most suitable representation for analyzing PET images because of connectivity restrictions [43]. Here, we extend polygon meshes to general graphs by relaxing the connectivity restrictions. Such graphs are easier to construct and are flexible enough to capture metabolic information. We construct a weighted network by encoding the metabolic information through an adjacency matrix $W=\{w_{ij}$}. The node corresponds to the brain regions, and the edge corresponds to the interregional correlation of brain metabolism. Specifically, the region parcellation in brain imaging is usually defined based on an anatomical atlas. In this study, we applied a predefined atlas, an automated anatomical labeling atlas with 90 (AAL-90) regions of interests (ROI) [28]. Once an ROI is specified, an overall summary measure within it can be calculated to assess the response as a whole, rather than on a voxel-by-voxel basis (step 2 of Figure 7). The most straightforward way to do so is by taking the average standard uptake values (SUV) of all voxels within the ROI. The SUV of a specific ROI is $\frac{1}{M}\overset{M}{\underset{p\;=\;1}{\sum }}v_{p}$, where *M* is the total voxel number in a given ROI and $v_{p}$ is the FDG uptake value of voxel *p*. Given *K* subjects and *N* brain regions, let $SUV_{i}\;=\;\left\{SUV_{i1},SUV_{i2},\ldots ,SUV_{iN},\right\}\left(1\leq i\leq N\right)$ be the vector of average SUV in *i*-th ROI of all *K* subjects (step 3 of Figure 7), and the edge weight $w_{ij}$ between two brain regions is defined as 1-Pearson correlation of SUV between them (step 4 of Figure 7), i.e.

(1) $$w_{ij}\;=\;1-\frac{cov\left(SUV_{i},SUV_{j}\right)}{\sigma_{SUV_{i}}\sigma_{SUV_{j}}},$$

where $SUV_{i},SUV_{j\;}$ are the average SUV in *i*-th and *j*-th brain region respectively, *cov* is the covariance, σ is the standard deviation, and $\frac{cov\left(SUV_{i},SUV_{j}\right)}{\sigma_{SUV_{i}}\sigma_{SUV_{j}}}$ is coefficient of Pearson correlation.

### 4.4. Network Indices

In clinical settings, doctors prefer single indices as biomarkers because a single neuroimaging index provides a practical reference for evaluating disease progression and for effective treatments. Generally, there are some available network indices based on graph theory that measure brain global attributes. In addition, we focus on some univariate indices that were developed from persistent homology in algebraic topology, and compare them with the network indices from traditional graph theory in our experiments.

#### 4.4.1. Traditional Graph Theory Indices

Traditionally, graph theoretical analysis has been applied to measure brain network topological features. In this study, three global network indices based on graph theory are investigated, including characteristic path length (CPL) [6], network diameter (ND) [9], and modularity (Mod) [7].

Briefly, CPL can be understood as indicating a network with “easily” transferred information. It is the average shortest path length between all pairs of nodes in the graph, and is calculated as $CPL=\frac{1}{N\left(N-1\right)}\underset{i\in V,j\in V,i\neq j}{\sum }d_{i,j}$, where *d_i_*,*_j_* is the shortest path length between nodes *i* and *j*. Note that infinitely long paths (i.e., paths between disconnected nodes) are not included in computations. ND is the greatest distance between any pair of nodes, and is defined as $ND=\underset{i\in V}{max}\underset{j\in V}{max}d_{i,j}$. It enables understanding of the size of a network. A graph with a large ND and small CPL would therefore be considered an efficient network. Mod describes the extent to which a network has modules that differ from others, each of which is independent and functionally specialized [7]. Computationally, it is expressed as $Mod=\underset{i\in M}{\sum }\left[c_{ii}-{\left(\underset{j\in M}{\sum }c_{ij}\right)}^{2}\right]$, where *i* and *j* are individual modules in the set of all modules *M*, and *c* is the proportion of existing connections between two modules.

In practice, we filtered the weighted network before computing these graph-based indices by only selecting the edges whose corresponding *p*-values passed through a statistical threshold (Bonferroni corrected *p* < 0.05) and then adopted the Brain Connectivity Toolbox (https://sites.google.com/site/bctnet/) [7] for their implementation (step 6 (right) of Figure 7).

#### 4.4.2. Persistent Features Based on Persistent Homology

Persistent homology is an emerging mathematical concept for characterizing shapes in complex data, and the persistence features based on BNP are widely recognized as a useful feature descriptor. BNPs can distinguish robust and noisy topological properties over a wide range of graph filtrations based on the connectivity of *k*-dimensional simplicial complexes [18] (step 6 (left) of Figure 7). Graph filtration is an important tool [24] in persistent homology that constructs nesting subnetworks in a coherent manner and avoids thresholding selection (step 5 of Figure 7). BNPs have been successfully applied to measure brain networks based on FDG-PET and structural MRI data [23] in some neurodegenerative diseases. In our previous study [25], we proposed an integrated persistent feature (IPF) by integrating an additional feature of connected component aggregation cost with BNP to achieve holistic descriptions of graph evolutions. The IPF at filtration $\lambda_{i}$ is defined as [25]

(2) $$IPF_{\lambda_{i}}=\{\begin{array}{c} \frac{m-i}{m\left(m-1\right)}\overset{m-1}{\underset{k=i+1}{\sum }}\lambda_{k},\;0\leq i\leq m-2\\ \;0,\;i=m-1\; \end{array},$$

where the maximal graph filtration is $\lambda_{0}=0<\lambda_{1}<\lambda_{2}<\;\cdot \cdot \cdot \;<\lambda_{m-1}$. As the IPF plot over all possible filtration values is a monotonically decreasing convergence function, the absolute value of the slope of IPF plot (SIP) was defined as a univariate network index and was successfully applied to quantify brain network dynamics on rs-fMRI data of AD. Both the BNP and SIP indices indicate the rate of connecting components converging over the filtration value, and can be thought as the information diffusion rate or the convergence speed with said network.

#### 4.4.3. The Kernel-Based IPF (KBI) Index

Although the SIP has been developed as a univariate network index in our previous study, it may not be the most appropriate way to describe IPF plot as it is nonlinear, strictly speaking. Recently, some kernel methods [26,27] have been defined on persistent homology to measure the distance between persistence diagrams, which are not only provably stable but also discriminative. Therefore, we employ the framework of kernel embedding of the IPF plot into reproducing kernel Hilbert spaces [26]. Given a point set of IPF plot $X=\left\{x_{1},x_{2},\cdots x_{N}\right\}$ and a template $T=\left\{t_{1},t_{2},\cdots t_{N}\right\}$ that are obtained from an average metabolic network of all NC subjects, the kernel-based IPF (KBI) index is defined as

(3) $$KBI\left(X\right)=\frac{1}{N}\overset{N}{\underset{x_{i}\in X,\;t_{i}\in T,\;i=1}{\sum }}{tan}^{-1}(C\left(\lambda_{i}^{X}{)}^{p}\right)\;{tan}^{-1}(C\left(\lambda_{i}^{T}{)}^{p}\right)e^{-{\frac{x_{i}-t_{i}}{2\sigma^{2}}}^{2}},$$

where ${tan}^{-1}(C\left(\lambda_{i}^{X}{)}^{p}\right)$ and ${tan}^{-1}(C\left(\lambda_{i}^{T}{)}^{p}\right)$ are both increasing functions with respect to maximal graph filtrations $\lambda^{\left(X\right)}\;\mathrm{of}\;X$ and $\lambda^{\left(T\right)}\;\mathrm{of}\;T$, and are used for weighting the persistence (λ is a sequence of persistence of zeroth homology in fact). Hence, an essential persistence gives a large weight and a noisy persistence produces a small weight. By adjusting the parameters *p*, σ, and *C*, we can control the effect of the persistence. In our practice, we set *p* = 5,

(4) $$\sigma =\underset{\forall X}{median}\{\underset{x_{i},x_{j}\in X,\;i<j}{median}\|x_{i}-x_{j}\|\},$$

(5) $$C={\left(\underset{\forall \lambda^{X}}{median}\{\underset{\lambda_{i}^{X}\in \lambda^{X}}{median}\left(\lambda_{i}^{X}\right)\}\right)}^{-p},$$

so that they take close values to many $\left\|x_{i}-t_{i}\right\|$ and $\lambda_{i}^{X}$, respectively.

### 4.5. Statistical Analysis

We applied a group-wise statistical analysis of permutation test to all network indices in the last section between AD, MCI, and NC groups. As there are no prior studies on the statistical distribution of any network index, it is difficult to construct a parametric test procedure. Moreover, as there is only one group-wise network for each group, it is necessary to empirically construct the null distribution and determine the *p*-value. The steps of our permutation test are described as follows. First, the actual network index difference in means between two groups is calculated according to the actual grouping of their subjects. Second, the subjects are randomly assigned to two groups, each of which is assigned the same group size. We then construct two group-wise networks based on such permutated groups and recalculate their indices, whose difference is recorded. This process is repeated 10,000 times and 10,000 permuted differences are obtained. Finally, the total number of permuted differences larger than the actual difference is counted and divided by 10,000. The obtained value is the probability of no difference between the groups, that is, the *p*-value.

### 4.6. Classification

We evaluate the power of our method by classification analysis. In this study, our proposed KBI index and other comparison network indices have only one univariate feature to discriminate the global brain network structure. Since the samples are limited, we apply resampling technology beforehand. For a specific group, half subjects are removed randomly at a time and the remaining subjects are used to construct a group-wise network. Resampling all subjects for *n* times repeatedly in each group until the results are stable, we can obtain *n* group-wise networks. Each group-wise network is an average of all involved subjects graphs. In our practice, the number of resampling times is set to 5000 (*n* = 5000) and we obtain 5000 resampled networks for each group. Each network can yield a KBI index and other comparable network indices. Then, we compute the values of proposed KBI index, as well as other network measures for all resampled networks, and run support vector machine (SVM) [44] on them. We conduct leave-one-out crossvalidation experiments to evaluate the classification performance. The classification accuracy, sensitivity (i.e., true positive rate), specificity (i.e., true negative rate), and area under the curve (AUC) of receiver operating characteristic (ROC) are severed as criterion of classification performance.

## 5. Conclusions

This work proposed a novel network index KBI based on our prior work of persistent feature IPF to measure the metabolic brain network of FDG-PET on cognitively impaired cohort. The proposed KBI encoded a great deal of dynamic information over all possible scales that may be inaccessible by standard graph-based measures. Compared to previous slope-based approaches of persistent homology, our kernel-based network index is more accurate regarding the characterization of differences between persistent features. Our current results show that the slope of IPF plot present a pattern AD < MCI < NC in a FDG-PET cohort, consistent with our prior finding in an rs-fMRI cohort, and indicate a slower network integration rate in AD dementia and MCI. Moreover, the enhanced measurement KBI greatly boosted the performance of prior persistent features and outperformed some standard graph-based network indices in both statistical inference and classification experiments, suggesting that our method may serve as a valuable preclinical AD imaging biomarker.

## Figures and Tables

**Table 1:** The network index values of three groups.

Cohort	KBI	SIP	BNP	CPL	ND	Mod
AD	0.577	0.790	268.7	1.20	1.601	1.289
MCI	0.826	0.842	265.2	1.17	1.457	0.770
NC	0.988	0.882	245.5	1.15	1.436	1.004

**Table 2:** Statistical *p*-values for between-group differences by different graph indices on an AAL-90 atlas.

Cohort	KBI	SIP	BNP	CPL	ND	Mod
AD vs. MCI	**0.017**	0.036	0.048	0.058	0.397	0.074
AD vs. NC	**0.002**	0.042	0.054	0.285	0.266	0.063
MCI vs. NC	0.036	0.076	0.339	0.062	**0.031**	0.458

Only the proposed KBI index detected significant difference in any between-group (any *p*-value < 0.05).

**Table 3:** Statistical *p*-values for between-group differences of KBI by different connectivity definitions.

Between-Group	Definitions of Distance Function			
	Kendall Correlation	Spearman Correlation	Partial Least Squares	Granger Causality Modeling
AD vs. MCI	**0.003**	0.007	0.159	0.399
AD vs. NC	0.005	**0.003**	0.047	0.375
MCI vs. NC	**0.109**	0.224	0.198	0.238

**Table 4:** Statistical *p*-values for between-group difference of different network indices on HOA-110 atlas.

Between-Group	KBI	SIP	BNP	CPL	ND	Mod
AD vs. MCI	**0.022**	0.032	0.063	0.092	0.424	0.035
AD vs. NC	**0.013**	0.053	0.033	0.458	0.290	0.081
MCI vs. NC	**0.042**	0.051	0.242	0.088	0.076	0.404

Similarly, only the proposed KBI index detected significant difference in any between-group.

**Table 5:** Statistical *p*-values for between-group differences by specifying Pearson correlation as connectivity directly.

Between-Group	CPL	ND	Mod
AD vs. MCI	0.214	0.040	0.081
AD vs. NC	0.063	0.083	0.173
MCI vs. NC	0.004	0.281	0.005

**Table 6:** Demographic information of the subjects in this study.

	AD (*n* = 140)	MCI (*n* = 280)	NC (*n* = 280)	*p-*value *^c^*
Age *^a^*	74.2 ± 7.8	73.9 ± 8.0	75.0 ± 6.6	0.20
Gender *^b^*	70/70	140/140	140/140	1
CDR	≥1	0.5	0	--

Key: AD, Alzheimer’s disease; MCI, mild cognitive impairment; NC, normal control; ^a^ mean ± SD; ^b^ male/female number; ^c^ statistical group significance using ANOVA test.
